# An Expert Consensus Study Regarding Management Practices to Prevent Infectious Mortality in Preweaned Beef Calves in Western Canada

**DOI:** 10.3390/vetsci11100453

**Published:** 2024-09-25

**Authors:** Virginia Margarita Sanguinetti, Cindy Adams, John Campbell, Sylvia L. Checkley, Claire Windeyer

**Affiliations:** 1 Faculty of Veterinary Medicine, University of Calgary, Calgary, AB T2N 4N1, Canada; virginia.sanguinetti@ucalgary.ca (V.M.S.); cadams@ucalgary.ca (C.A.); slcheckl@ucalgary.ca (S.L.C.); 2 Western College of Veterinary Medicine, University of Saskatchewan, Saskatoon, SK S7N 5B4, Canada; john.campbell@usask.ca

**Keywords:** best management practices, disease control, risk factors, health management, calf survival, Delphi method

## Abstract

**Simple Summary:**

Preventing preweaning disease is essential to minimize calf mortality in cow–calf herds. However, there is limited evidence concerning the best management practices to achieve this. Given this, expert opinions were used to fill this knowledge gap. The first objective was for a group of veterinarians to determine which practices are most useful in preventing calf mortality in herds, considering their effectiveness, ease of implementation, and economic feasibility. A second objective was for them to define practices that should be included in a decision tool to facilitate discussions between producers and veterinarians. The Delphi consensus-building method was used to collect expert opinions through two questionnaire rounds and two workshops. Twelve veterinarians were recruited to participate. They considered the effectiveness of a practice more important than its ease of implementation and economic feasibility. Administering clostridial vaccines and providing colostrum to calves that required it were considered practices that were ‘always useful for all herds’. Twenty-five additional practices were considered at least useful ‘very much for some herds’. Antibiotic administration was considered the least useful. However, all practices were considered relevant enough to be included in the future tool. Therefore, the findings of this study will be used in a tool that will facilitate discussions and enable the implementation of best management practices.

**Abstract:**

Disease prevention is a cornerstone of herd management for minimizing preweaning calf mortality. However, scientific evidence about the usefulness of practices in herds is scarce. The first objective was for a group of veterinarians to determine which practices are most useful considering their effectiveness, ease of implementation, and economic feasibility. A second objective was for them to define which practices should be included in a tool to facilitate discussions between producers and veterinarians. Expert opinions and consensus were determined using a modified Delphi approach. During two questionnaire rounds, participants scored the effectiveness, ease of implementation, and economic feasibility of each practice. Overall scores for each practice were calculated, and feedback reports were sent to participants between rounds showing the groups’ median responses. Consensus on which practices should be included in the tool was targeted during the workshops. Twelve veterinary experts participated. Administering clostridial vaccines and providing calves with colostrum in case they had not nursed were considered practices that were ‘always useful for all herds’. However, most practices had intermediate levels of usefulness, and among these, antibiotics were considered the least useful. Nevertheless, all practices discussed during the workshops attained a consensus about being included in the future tool to facilitate on-farm discussions.

## 1. Introduction

Infectious mortality in preweaned beef calves affects cow–calf herds’ productivity and economic success [1,2]. In western Canada, calf mortality from 24 h to weaning was estimated at 1.5% (95% CI: 0–10.4%) for calves born to heifers and 1.9% (95% CI: 0–6.9%) for those born to cows [3]. In this production system, weaning is typically carried out at 7 months of age [4]. Significant causes of calf mortality include neonatal calf diarrhea (NCD) and bovine respiratory disease (BRD) [5]. Mortality attributed NCD and BRD was estimated at 11.9% (±3.2% SE) and 8.1% (±2.3% SE), respectively [5]. Nonetheless, in North America, the number of calves affected, as well as those that die, vary widely between years and across herds [2,3,5,6,7]. Therefore, given their potential impact on cow–calf operations, best management practices are essential to minimize detrimental effects of calfhood disease and mortality.

In herds, the use of best management practices may potentially reduce the risk of disease and mortality [8]. This can be achieved by implementing practices that target disease directly, such as biosecurity [9] and vaccination [10], or with practices that work indirectly by limiting the impact of predisposing risk factors, such as reducing stress and minimizing stocking density [11,12,13,14]. However, the usefulness of many such practices is still unknown. A systematic review that is underway by these authors will summarize the evidence of practices having statistically significant protective associations [15]. However, statistical associations are not the same as on-farm usefulness. Given this, there remains a knowledge gap regarding which practices are most useful for preventing calf mortality in cow–calf herds.

Consensus among experts is considered a more reliable source of evidence than experts’ individual opinions [16]. A commonly used consensus-building approach is the Delphi method [17]. The process involves sequential rounds of questionnaires, indirect communication between experts through controlled feedback reports, and targeting consensus, which may or may not be attained [18,19]. This method has recently been used in veterinary medicine to fill other knowledge gaps in animal welfare and One Health [20,21].

The objective of this study was for a group of veterinarians to first identify which practices are most useful for preventing calf mortality in herds, considering their effectiveness, ease of implementation, and economic feasibility. A second objective was to determine which practices should be included in a tool to facilitate discussions between producers and veterinarians.

## 2. Materials and Methods

### 2.1. Ethics and Reporting

The University of Calgary Conjoint Faculties Research Ethics Board approved this study (REB21-0295). Reporting followed the Guidance on Conducting and REporting DElphi Studies (CREDES) recommendations [22].

### 2.2. Selection of Experts

A modified Delphi method was used to collect expert opinions [16,18,23,24]. Potential participants were selected based on meeting specific education and experience criteria. Eligibility criteria included being a Doctor in Veterinary Medicine and being judged by the research team to have working knowledge of the western Canadian cow–calf management system. The principal investigator’s network was used to select members of academia and private practitioners. The recruitment process involved contacting potential participants individually and explaining the study’s objectives and other relevant characteristics (e.g., anonymity during the questionnaire rounds and the right to withdraw from the study at any time). A recruitment letter and consent form were sent to document their written consent to participate in the study. If they declined to participate, they could suggest another potential participant to be contacted. Additional participants were recruited to pilot the questionnaires.

### 2.3. Management Practices and Outcomes Assessed

The management practices and outcomes assessed were based on a systematic review of the literature [15]. The practices assessed included colostrum management, breeding, calving, nutrition, pasture management, biosecurity, vaccination, and antibiotic use. Practices could be related to pregnant dams or preweaned calves. The outcomes were preweaning mortality, fatal NCD, and fatal BRD. Preweaning mortality was defined as “all calf deaths, regardless of whether a specific cause of death was determined, that occur from 24 h after birth until the calves are weaned”. It was stated that, for the purposes of this study, this did not include abortions, stillbirths, or perinatal deaths at <24 h of age, nor did it refer to mortality in post-weaned calves or mature animals within the herd. Fatal NCD was defined as “deaths that occur as a result of NCD (i.e., scours)” and additional information was provided about the disease [25]. Fatal BRD was defined as “deaths that occur because of infectious BRD,” and similarly, information about BRD was provided [26,27]. Most of the practices included in the study were directed towards preweaning mortality; however, a subset of practices that focused on fatal NCD or fatal BRD were also included.

### 2.4. Questionnaires

First, participants were asked to assign, in general terms, a relative weight to the effectiveness, ease of implementation, and economic feasibility of practices as a proportion of 100%. Definitions were provided for each of these elements. Effectiveness referred to “whether a management strategy is likely to do more good than harm and to prevent clinical cases when provided under usual circumstances” [28]. Ease of implementation referred to “a lack of difficulty in integrating a new management strategy into current activities”. Economic feasibility was defined as “the financial justification for an implemented action to prevent detrimental effects regarding animal health issues” [29]. Subsequent questions required participants to score each of these elements for specific practices using a defined scale. A score of 0 indicated that the practice was considered either effective, easy to implement, or economically feasible ‘not at all for any herd’, 1 indicated ‘not at all for most herds’, 2 indicated ‘somewhat for some herds’, 3 indicated ‘very much for some herds’, 4 indicated ‘very much for most herds’, and 5 indicated ‘always for all herds.’ Additional questions required participants to select their vaccine preferences by checking boxes. Free text spaces were provided if participants wanted to add practices or comments. The content of the first and second questionnaires was very similar except for the addition of three practices to the second questionnaire that were suggested by participants through free-text answers within the first questionnaire.

### 2.5. Data Management of Questionnaires and Feedback Reports

Prior to data analyses, participants’ responses were anonymized using an alphanumerical code. Analyses were conducted using Microsoft Excel (Microsoft Corporation, Redmond, WA, USA). For questions requiring participants to input percentages or scores, the median, minimum, and maximum percentages or scores were reported for each practice. Also, overall usefulness scores were determined for each practice. These were calculated using the relative weight of effectiveness, ease of implementation, economic feasibility determined from the first questionnaire, and the median scores of each element for each practice. For questions requiring participants to select vaccines of preference, thresholds to determine ‘low’, ‘medium’, and ‘high’ levels of preference were determined using the 25th and 75th percentiles of the responses given.

Feedback reports were created, showing the participants’ aggregated responses to each question. These were sent to participants after each questionnaire round. Within these reports, colour coding was used to help visualize median scores of individual practices (e.g., between 0 and 1 cells was shaded red, between 2 and 3 yellow, and between 4 and 5 green). Also, colour coding was used to show the results of vaccine preference. Vaccines with ‘low’ preference were shaded red, ‘medium’ were yellow, and ‘high’ were green. The overall usefulness of practices was only shown in the second feedback report. Practices were listed in descending order of usefulness.

The participants’ responses on the overall usefulness of practices were deemed stable throughout questionnaire rounds if the median scores did not change from the one-point categories within the numerical scale (i.e., between 0 and 1, 2 and 3, or 4 and 5) [16,30], meaning that their usefulness in herd categorization did not change. Potential areas of controversy or uncertainty were identified based on the range of scores given for each practice during the first questionnaire. The criteria used to define a wide range of scores involved having at least a four-point difference between the minimum and maximum scores given by participants (e.g., 0–4, 1–5, 0–5); otherwise, the range of scores was defined as a narrow one. Furthermore, responses with wide ranges identified during the first questionnaire were compared to those with wide ranges of scores during the second questionnaire. The dynamic of the participants’ responses for these areas was described, showing those that increased, decreased, or whose ranges of scores remained the same during questionnaire rounds [31,32].

### 2.6. Workshops and Data Management

Two 120-minute-long workshops were scheduled on different dates to ensure that as many participants could attend as possible. These were held online using an online platform (Zoom Cloud Meetings, Zoom Video Communications, Inc., San Jose, CA, USA). Two researchers (CW and VMS) moderated the sessions. The objective was to answer the question, “Which management practices should be included in a decision tool to be used on a farm?” First, participants were required to independently vote on which practices they would include in a decision tool. Only practices considered at least useful ‘very much for some herds’ during the second questionnaire were offered. Before voting, explanations about the intended use of the decision tool and thresholds used to determine which practices would attain consensus (i.e., at least 60% positive votes [33]), semi-consensus (i.e., between 40 and 59% positive votes), and negative consensus (below 39% positive votes) with respect to a practice being included were defined [34]. A confidential voting process was conducted in Qualtrics (Provo, UT, USA). Voting results were downloaded into an Excel spreadsheet (Microsoft Corporation, Redmond, WA, USA). These were analyzed in real-time, and colour coding was used to show participants which practices achieved consensus (green), semi-consensus (yellow), and negative consensus (white). Practices that attained consensus were automatically considered relevant to be included in the tool. Those that achieved semi-consensus or negative consensus were discussed and could be included in the tool if participants agreed. During the discussion, moderators ensured that all participants could disclose their opinions and that no participant dominated the session.

## 3. Results

### 3.1. Expert Demographics and Participation

Three participants were used to pilot and refine the first questionnaire, and 12 more were enrolled in the study. Only one participant declined involvement in this study. In total, 12 out of 12 participants completed the first questionnaire, 11 out of 12 the second, and 8 out of 12 participated in the workshops (workshop 1: n = 6, workshop 2: n = 2). Out of the 12 participants enrolled in the study, 5 were women (41.7%) and 7 were men (58.3%). Six were veterinary practitioners (50%), four were members of academia (33%), and two were both (17%). Ten graduated from veterinary programs in North America (83.3%) and two from other parts of the world (16.7%). The median graduation year was 1996 (range: 1972–2019). In addition to their veterinary degree, eight (66.7%) had some postgraduate degree or training (e.g., MSc, PhD, board certification).

### 3.2. Questionnaire Responses

During the first questionnaire, one participant did not submit scores on some practices, including vaccination to prevent BRD mortality, biosecurity, breeding, calving, nutritional, and pasture management (Question (Q)3a, Q6, and Q7; Supplementary Material S1). During the second questionnaire, one participant did not submit scores for some biosecurity practices (Q6; S2). Additionally, two scores given to breeding, calving, nutritional, and pasture management practices were excluded from the analysis because the participant wrote an invalid score (e.g., a score of 2.5 when only whole numbers were accepted).

In the first questionnaire, participants determined that the usefulness of a practice should be weighted as 50% (range: 20–70) for effectiveness, 22.5% (range: 10–33) for ease of implementation, and 27.5% (range: 15–50) for economic feasibility (Supplementary Material S1). In the second questionnaire, participants determined that the usefulness of a practice should be weighted as 50% (range: 35–70) for effectiveness, 25% (range: 15–30) for ease of implementation, and 25% (range: 10–40) for economic feasibility (S2).

The practices of administering clostridial vaccines to calves and feeding colostrum or colostrum replacer to the calf using a nipple bottle or oesophageal tube if it had not nursed were considered useful ‘always for all herds’. Most of the practices assessed, including breeding, calving, nutrition, and pasture management, biosecurity, and vaccination practices, had intermediate levels of usefulness (Table 1). Specifically, 12 were considered useful ‘very much for most herds’ and 13 ‘very much for some herds’. The least useful practices were deemed useful ‘somewhat for some herds’ and included metaphylactic and prophylactic use of antibiotics, vaccination of calves against NCD pathogens during the first week of life, and vaccination of dams at spring turnout against BRD pathogens. None of the practices assessed were considered useful ‘not at all for most herds’ or ‘not at all for any herd’.

Each practice’s overall median usefulness scores between questionnaire rounds were stable. None of the practices had a change of more than one point in their median scores; therefore, none changed their usefulness categorization (Table 1). However, participants gave a wide range of scores (e.g., 0–5, 1–5, 0–4) to 27 elements of the practices during the first questionnaire. From the first to the second questionnaire, the ranges of scores for 19 of these 27 elements decreased, eight remained the same, and one increased from a narrow range of scores to a wide range (Table 2). At the end of the second questionnaire, nine elements of practices still had wide ranges of scores. These included the effectiveness and economic feasibility of vaccination against BRD pathogens during pregnancy check, spring turnout, or pregnancy, and of calves during the first week of life, as well as the ease of implementation of asking about disease history before purchasing animals.

### 3.3. Workshops

Six participants attended the first workshop. During voting, 21 practices attained consensus, 4 semi-consensus, and 3 negative consensus. After the discussion, participants agreed that all practices were relevant enough to be discussed with producers, and thus should be included in the tool. Participants suggested that additional practices and risk factors should also be considered, including predation, assisted calving, genetic selection, and the use of toltrazuril. Comments were made about having difficulty assessing the effectiveness of practices using the scale provided due to the contextual nature of making recommendations about practices. Specifically, participants expressed a need for more details about the disease risk and mortality of the herd to score the effectiveness of practices with more certainty.

Two participants attended the second workshop and only one researcher moderated the session. During voting, 16 practices attained consensus, 9 semi-consensus, and 3 negative consensus. After the discussion, similar to what happened in the first workshop, participants agreed that all practices should be included in the tool. They also considered that other additional practices and risk factors should have been assessed. These included pre-breeding vaccination and the incidence of assisted calving in the herd. A thorough discussion took place concerning the extent to which veterinarians may influence certain practices (e.g., delaying the calving season, reducing stocking density, or the preference for feeding minerals to dams rather than administering to calves). Some discussion took place concerning the scale used, the approach of assessing practices individually rather than in the context of each other, and the types of herds or situations for which the future tool would be helpful.

## 4. Discussion

This study helped prioritize and compare the relative usefulness of practices to prevent calfhood disease and minimize calf mortality. Furthermore, it generated and collected specific evidence concerning the effectiveness, ease of implementation, and economic feasibility of practices. To the best of our knowledge, this is the first study that attempts to narrow this knowledge gap using expert opinions about this topic in Canada.

When considering the relative importance of elements in a practice’s overall usefulness, the participants prioritized its effectiveness over its ease of implementation and economic feasibility. A study assessing the producer adoption of digital technologies in agriculture also explored effectiveness, ease of implementation, and economic feasibility and found that their relative importance was very similar to the present study [35]. In contrast, an expert consensus study on disease control conducted in Switzerland only evaluated the effectiveness of practices [36], suggesting an investigation of the other elements was not considered to be important by those authors. The emphasis on effectiveness likely reflects the fact that the aim of implementing practices is to minimize or prevent disease on herds [37,38], and effectiveness is the element that best achieves this. Ease of implementation and economic feasibility may be given less importance because they are more related to implementing the practices rather than whether the practices achieve their intended purpose (i.e., preventing disease and mortality).

Vaccination of calves against clostridial pathogens was considered a useful practice for all herds. Similarly, representatives from different groups in western Canada, including veterinarians, also highly encouraged vaccination against clostridial pathogens [39]. However, within that study, the consensus of the recommendation that a vaccine should be considered as a core vaccine (i.e., one that protects herds from endemic diseases that pose a severe disease risk and should be administered annually [40]) varied depending on the clostridial species involved, from only 69% and 54% for *C. tetani* and *haemolyticum*, respectively, to 85 to 100% for *C. chauvioe*, *septicum*, *noyvi*, *perfringens* type D, and *sordelli*. It is well accepted that vaccination is a keystone in reducing clostridial diseases in ruminants worldwide [41,42,43]. However, scientific evidence for its effectiveness in preventing naturally occurring disease and mortality is scarce [42]. Nonetheless, a review reported that the level of protection given by vaccines was close to 100% in challenge studies conducted on ruminants [42]. Vaccination is a relatively quick practice to conduct and may be easily incorporated into other usual activities carried out on farm (e.g., branding). This practice is likely economically beneficial given that the average price of the vaccine is relatively low (CAD 0.50 to 1.68 per dose), the case fatality rate of clostridial diseases is high [43], and a calf that dies before weaning does not result in the expected revenue from selling it [44]. Still, a comprehensive economic study has not been conducted to prove this hypothesis. This practice is already common in Canada, with 87% of pre-weaned beef calves in herds being vaccinated. However, only 26% received a second dose [45], which does not align with general recommendations for core vaccination against clostridial pathogens [46]. Therefore, the use of this practice should be further encouraged in herds, and future studies are needed to tailor the optimum booster protocols and prove its economic benefit in herds.

Feeding colostrum or colostrum replacer to a calf using a nipple bottle or an oesophageal tube if it has not been nursed was also considered always useful for all herds. Colostrum has been described as the most important practice affecting calfhood health and mortality [47]. This is because calves are born with a naïve immune system and rely exclusively on colostral antibodies for protection against infectious diseases during their first weeks of life [48]. Similar to this study, herds that routinely intervened with colostrum in assisted calves at birth and in calves that had not nursed from their dam had significantly less mortality compared to those that did not do so [49]. This is probably because calves that require colostrum intervention are usually impacted by subjacent factors, such as difficult calving [50,51], and thus cannot nurse by themselves in a timely manner, affecting the transfer of passive immunity (TPI) [52]. The relationship between failed TPI and the increased risk of morbidity and mortality is well documented [53,54,55,56], and the estimated costs per calf with failed TPI was estimated at EUR 80 [54]. However, in beef herds, calves with inadequate TPI are more prevalent than those with failed TPI, and this has also been associated with increased mortality [52]. Furthermore, these calves may be more difficult to identify under field conditions than those with failed TPI, given that they usually nurse but do so insufficiently. Therefore, preventing subjacent causes that affect calves’ ability to nurse from the dam, promptly identifying calves at risk of both failed and inadequate TPI, and providing calves with colostrum are useful practices to minimize mortality in herds.

Most of the practices assessed in this study had intermediate levels of usefulness. These included breeding, calving, nutrition, pasture management, biosecurity, and many vaccination practices. The reason that most practices were classified useful ‘very much for most herds’ or ‘very much for some herds’ was likely because their effectiveness in preventing disease and mortality depends on context. During the piloting of the first questionnaire and throughout the workshop discussions, several participants mentioned having difficulties assessing individual practices without more context concerning the other practices used in the herd. They requested information about herd demographics and the use of other practices, particularly the level of intensive or extensive management of the herds to understand disease and mortality risk better [57]. This aligns with the idea that a practice’s effectiveness may vary with the risk of disease and mortality [58]. However, there is no reliable evidence proving that the effectiveness of these practices varies with the disease or mortality risk in cow–calf operations [59,60].

Antibiotic treatments used for both prophylaxis and metaphylaxis were among the lowest-scored practices. This suggests that the participants considered them less useful than other practices and that they should only be used in some herds in some situations. This aligns with initiatives that aim to reduce the use of antibiotics, and thus the potential risk of antibiotic resistance in humans, plants, and animals [61]. In cow–calf operations, the current use of antibiotics typically involves the treatment of BRD, NCD, navel infection, and arthritis [62]. A scoping review focused on North American cow–calf herds found that among studies that reported the intended use of antibiotics, five out of seven stated that besides treatment, antibiotics were also used to prevent or control disease [63]. However, most of these studies were conducted before an important Canadian regulation was implemented requiring that all medically important antimicrobials for human medicine be dispensed with a veterinarian’s prescription [64]. A Canadian study from 2020 showed that no herds were using class one antimicrobials, including fluoroquinolones, to prevent disease in calves, compared to 2014, when one herd reported using enrofloxacin for this purpose [65,66]. This suggests this is already a rare practice and is potentially decreasing given that the current regulation requires veterinarian prescriptions for disposing of these classes of antimicrobials. 

The median scores of the usefulness of the practices were stable between questionnaire rounds, and all practices attained consensus to be included in the future tool. This may have been associated with the consensus-seeking nature of the Delphi method used [17,67]. Participants were recruited from western Canada, where the tool is intended to be implemented. Therefore, given that they all had similar expertise and worked within the beef industry in the same region, their perceptions of practices may be similar. Additionally, aspects of this study’s design could have contributed to the stability of responses and consensus. The first round began with a structured questionnaire where the practices assessed were defined based on the findings of a systematic review [15] instead of an open-ended questionnaire where participants could decide on the practices to be assessed [16]. Other hypothetical reasons why all practices attained consensus include, despite the researchers envisioning the tool to focus exclusively on practices with a large impact on calfhood health, the fact that participants probably thought it was beneficial to discuss practices that also affected other important objectives, for example, optimizing the reproductive rates and weaning weights of calves. Therefore, even though some practices, for example, the mineral supplementation of dams, probably have a larger impact and more direct effect on reproductive rates [68] compared to calfhood health [69], they are still considered important topics to be discussed with producers and thus, attained consensus should be included in the tool.

Some practices were given a wide range of scores, potentially indicating areas of uncertainty or controversy between participants [31,32]. During the first questionnaire, these areas included the effectiveness of biosecurity practices such as vaccinating cattle before introducing them to the herd, asking about disease history, and disease testing before introducing purchased cattle to the herd, as well as the effectiveness and economic feasibility of dam vaccination against BRD. This uncertainty may arise from the scarcity of evidence regarding these practices in cow–calf herds. For example, most of the evidence concerning biosecurity comes from feedlot and dairy productive systems, which typically have a greater degree of commingling, a higher stocking density, and thus a higher disease and mortality risk compared to the comparatively extensive cow–calf system seen in western Canada [70,71]. Likewise, no studies or reviews have proved the clinical effectiveness or economic feasibility of dam vaccination against BRD in pre-weaned beef calves.

The dynamic of the ranges of scores during the second questionnaire for potential areas of uncertainty or controversy differed across practices. For biosecurity practices, the ranges became narrower, while for dam vaccination against BRD pathogens, they remained wide. This may indicate that for biosecurity practices the consensus-seeking nature dominated the response dynamic, so participants reconsidered their responses based on the groups’ median response, and therefore, the range of scores decreased [72]. In contrast, participants may have had conflicting views or strong opinions about dam vaccination against BRD pathogens that drove them not to change their responses from the previous questionnaire round [72]. Within the questionnaires’ free-text spaces and workshops discussions, participants indicated that pre-breeding dam vaccination against reproductive diseases should have also been included in this study. Only practices for pregnant dams or calves were included in this study. Yet, participants may have considered pre-breeding vaccines as relevant because these are the most commonly used vaccine in dams [45,73]. While the pre-breeding vaccination of dams is mainly intended to protect against reproductive diseases by targeting the viral agents bovine viral diarrhea virus, bovine herpesvirus type 1, bovine respiratory syncytial virus, and parainfluenza virus type 3, these pathogens are also involved in BRD in calves [74,75]. Calves born from vaccinated dams had higher antibody titers for BRD pathogens than those born from non-vaccinated dams [76]. Still, the impact of the timing of dam vaccination and BRD prevention in calves is unknown, likely contributing to the conflicting views on this practice’s usefulness.

Given that most practices were useful at intermediate levels and that participants requested additional details on context to score the practices more accurately, a potential limitation of this study was that practices were presented individually. This raised the question of whether practices should be grouped in packages in future studies of management practices. This concept refers to grouping a series of risk factors and management practices associated with, for example, one event, such as calving, and assessing their overall impact together rather than independently, which would potentially allow participants to have a sense of context and thus score practices more accurately.

## 5. Conclusions

This expert consensus study helped fill an important knowledge gap concerning best management practices in western Canada. Vaccination of calves against clostridial pathogens and providing colostrum to calves that had not been nursed were considered ‘always useful for all herds.’ Most breeding, calving, nutrition, pasture management, biosecurity, and vaccination practices were considered at least useful ‘somewhat for some herds’, and experts agreed all should be considered during discussions with producers about on-farm management practices. These findings and the subsequent decision tool will help cow–calf producers implement best management practices to minimize infectious disease and prevent calf mortality within their herds.

## Figures and Tables

**Table 1:** Overall usefulness of management practices, considering effectiveness, ease of implementation, and economic feasibility, assessed in an expert consensus study of practices to prevent infectious preweaning calf mortality in western Canadian beef herds.

Management Practices	Questionnaire 1	Questionnaire 2	Usefulness
Administering clostridial vaccines in pre-weaned calves	4.66	4.75	Always for all herds
Feeding colostrum or colostrum replacer to the calf using a nipple bottle, if it has not nursed	4.53	4.5	Always for all herds
Feeding colostrum or colostrum replacer to the calf using an oesophageal tube, if it has not nursed	4.55	4.5	Always for all herds
Administering clostridial vaccines to pregnant dams	4.28	4.25	Very much for most herds
Asking about the disease history of an animal or its herd prior to purchase	4.00	4.00	Very much for most herds
Calving heifers in a separate area or at a different time than cows	4.05	4.00	Very much for most herds
Forced feeding mineral supplementation to cows or heifers (e.g., mixing into feed)	3.66	4.00	Very much for most herds
Vaccinating calves at spring processing (i.e., ~1–3 months of age) *	4.00	3.75	Very much for most herds
Vaccinating new cattle prior to introducing them into the herd	3.78	3.75	Very much for most herds
Repeat purchasing from trusted sources	***	3.75	Very much for most herds
Limiting the breeding season length of 45–60 days	3.78	3.75	Very much for most herds
Segregating calves by age using pasture management, such as the sandhills or foothills calving systems	3.89	3.75	Very much for most herds
Moving cow herd from wintering area to a clean calving area	3.89	3.75	Very much for most herds
Isolating new cattle for a period of time prior to introducing them into the herd	3.55	3.50	Very much for most herds
Having a creep calf area for calves (e.g., creep feeding, calf shelters)	3.50	3.50	Very much for most herds
Limiting the gathering and bunching of the herd	***	3.25	Very much for some herds
Limiting the number of purchased animals	***	3.13	Very much for some herds
Parenteral vaccines against NCD ^a^ given to pregnant heifers **	3.00	3.00	Very much for some herds
Parenteral vaccines against NCD given to pregnant cows **	2.75	3.00	Very much for some herds
Using low stocking density, such as those associated with more extensive systems	3.11	3.00	Very much for some herds
Injectable or oral mineral supplementation given to calves	3.00	2.90	Very much for some herds
Vaccinating heifers and cows at pregnancy check against BRD ^b^*	2.73	2.75	Very much for some herds
Vaccinating pregnant heifers and cows against BRD *	3.00	2.75	Very much for some herds
Vaccination of calves within the first week of life against BRD *	2.78	2.75	Very much for some herds
Injectable mineral supplementation given to cows or heifers	2.75	2.75	Very much for some herds
Mass antibiotics treatment	2.78	2.75	Very much for some herds
Participating in certification programs (e.g., VBP+ ^c^)	3.00	2.60	Very much for some herds
Testing for disease prior to introduction to the herd (e.g., BVDV ^d^ or Johne’s)	2.89	2.5	Very much for some herds
Metaphylactic use of antibiotics	2.48	2.25	Somewhat for some herds
Parenteral vaccine given to calves during the first week of life (e.g., Bovine Rotavirus and Coronavirus modified live virus) **	2.25	2.00	Somewhat for some herds
Vaccinating heifers and cows at spring turnout against BRD *	2.28	2.00	Somewhat for some herds
Prophylactic use of antibiotics	1.73	1.75	Somewhat for some herds

^a^ Neonatal calf diarrhea; ^b^ bovine respiratory disease; ^c^ verified beef production plus; ^d^ bovine viral diarrhea virus. * The outcome in question was fatal BRD; ** The outcome in question was fatal NCD; *** Management practices suggested by the participants during the first questionnaire that were added to the second questionnaire.

**Table 2:** Management practices given wide ranges ^a^ of scores by participants during at least one of two sequential questionnaires in an expert consensus study of practices to prevent infectious preweaning calf mortality in western Canadian beef herds.

Management Practice		Effectiveness	Ease of Implementation	Economic Feasibility
Vaccination against NCD ^b^ pathogens	Pregnant dams		↓	
Vaccination against BRD ^c^ pathogens	Dams at pregnancy check	=		=
	Dams at spring turnout	=	↓	=
	Pregnant dams	=		=
	Calves within the first week of life	↑	↓	=
Vaccination against Clostridium pathogens	Pregnant dams	↓	↓	↓
Biosecurity	Vaccinating cattle prior to introducing to herd	↓	↓	↓
	Asking about disease history prior to purchase	↓	=	
	Disease testing prior to introduction			↓
	Participating in certification programs	↓		
Breeding, calving, nutrition, and pasture management	Injectable mineral supplementation given to cows or heifers	↓		
	Injectable or oral mineral supplementation given to calves	↓		
Antibiotic administration	Prophylactic use of antibiotics	↓	↓	↓
	Metaphylactic use of antibiotics	↓		
	Mass antibiotic treatment			↓

^a^ A wide range was defined as at least a four-point difference between the minimum and maximum scores given by participants (e.g., 0–4, 1–5, 0–5); ^b^ neonatal calf diarrhea; ^c^ bovine respiratory disease; ↓ elements whose ranges decreased in the second questionnaire compared to the first one (from wide to narrow range); ↑ elements whose ranges increased in the second questionnaire compared to the first one (from narrow to wide range); = elements whose ranges of scores remained the same during the second questionnaire compared to the first one.

## Data Availability

Dataset is not available due to requirements for participant anonymity.

## Supplementary Materials

The following supporting information can be downloaded at: https://www.mdpi.com/article/10.3390/vetsci11100453/s1, Supplementary material S1—Feedback report of the first questionnaire in an expert consensus study regarding management practices to prevent infectious mortality in preweaned beef calves in western Canada and Supplementary material S2—Feedback report of the second questionnaire in an expert consensus study regarding management practices to prevent infectious mortality in preweaned beef calves in western Canada.
