# Supplementary material for: An Expert Consensus Study Regarding Management Practices to Prevent Infectious Mortality in Preweaned Beef Calves in Western Canada
Source: Vet Sci. 2024 Sep 25;11(10):453. doi: 10.3390/vetsci11100453 (PMC11512374; doi:10.3390/vetsci11100453)
Supplement: Supplementary file 1 [file vetsci-11-00453-s001.zip › vetsci-3177188-supplementary.pdf]

Supplementary Material S1. Feedback report of the first questionnaire in an expert consensus study regarding management practices to prevent infectious mortality in preweaned beef calves in western Canada.

This is the first report showing the responses to Questionnaire 1.

The aim of this report is to provide feedback to the experts regarding the group's responses to Questionnaire 1. This is an essential step in the Delphi method where the experts (i.e. you) can reconsider your responses before answering Questionnaire 2.

How were responses analyzed?

For the question that required the input of percentages (Q1), the median, minimum, and maximum values were reported.

To analyze the scores for each element of a strategy (e.g., effectiveness, ease of implementation and economic feasibility) (Q2a, Q3a, Q4, Q5, Q6, Q7, and Q8), the median, minimum, and maximum scores are reported. Then, colour coding was used to visualize median scores, as shown below.

0-0.5	0.5-<1.5	1.5-<2.5	2.5-<3.5	3.5-<4.5	4.5-5
Not at all for any herd	Not at all for most herds	Somewhat for some herds	Very much for some herds	Very much for most herds	Always for all herds

To analyze questions that required experts to select a vaccine of choice (Q2b and Q3b), the percentage of respondents who selected each vaccine was calculated. Thresholds used to classify 'High', 'Intermediate', and 'Low' values were determined according to the 25th and 75th percentiles of the cumulative percent of responses. Colour coding was used to visualize 'High' (green), 'Intermediate' (yellow), and 'Low' (red) percents.

RESULTS:

1. Given your knowledge and experience, what is the relative weight that should be given to the following elements of a disease control strategy in general terms?

	Median % (min-max)
Effectiveness	50 (20-70)
Ease of implementation	22.5 (10-33)
Economic feasibility	27.5 (15-50)

VACCINATION:

2a. Vaccination is a disease control strategy that can be used to prevent fatal NCD in preweaned beef calves. **What is the effectiveness, ease of implementation, and economic feasibility of vaccinating each of the production groups listed below to reduce the incidence of fatal NCD?**

	**Effectiveness** (Median, min-max)	**Ease of implementation** (Median, min-max)	**Economic feasibility** (Median, min-max)
Parenteral vaccine given to calves during the first week of life (e.g. Bovine Rotavirus and Coronavirus modified live virus)	2 (1-3)	2.5 (1-3)	2.5 (1-3)
Vaccinating pregnant heifers and cows	3 (2-4)	3 (1-5)	3 (2-4)
Parenteral vaccines given to pregnant cows following label instructions	2.5 (2-4)	3 (1-5)	3 (2-4)

2b. Given your experience, what vaccine would you recommend for pregnant heifers and cows prior to the calving season to reduce the incidence of fatal NCD in preweaned beef calves?

Thresholds used for colour coding responses:

Categories	Percent of respondents
LOW	≤14%
INTERMEDIATE	14-48%
HIGH	48%

	Vaccine of choice	Percent of respondents who selected the vaccine
Parenteral vaccines given to pregnant heifers	Modified live Bovine Rotavirus and Coronavirus vaccine	8%
	Killed Bovine Rotavirus and Coronavirus	25%
	Killed Bovine Rotavirus and Coronavirus vaccine with *Clostridium perfringens* Type C & D and *Escherichia coli* bacterin-toxoid	92%
	Bacterin toxoid *Clostridium chauvoei*, *C. septicum*, *C. haemolyticum*, *C. novyi*, *C. sordellii*, *C. perfringens* Types C & D	33%
Parenteral vaccines given to pregnant cows	Modified live Bovine Rotavirus and Coronavirus vaccine	8%
	Killed Bovine Rotavirus and Coronavirus	17%
	Killed Bovine Rotavirus and Coronavirus vaccine with *Clostridium perfringens* Type C & D and *Escherichia coli* bacterin-toxoid	92%
	Bacterin toxoid *Clostridium chauvoei*, *C. septicum*, *C. haemolyticum*, *C. novyi*, *C. sordellii*, *C. perfringens* Types C & D	33%

3a. Vaccination is a disease control strategy that can be used to prevent fatal BRD in preweaned beef calves. **What is the effectiveness, ease of implementation, and economic feasibility of vaccinating the production groups listed below to reduce the incidence of fatal BRD in calves?**

	**Effectiveness** (Median, min-max)	**Ease of implementation** (Median, min-max)	**Economic feasibility** (Median, min-max)
Vaccinating heifers and cows at pregnancy check	2 (1-5)	4 (2-5)	3 (1-5)
Vaccinating heifers and cows at spring turnout	2 (0-5)	2 (0-4)	3 (0-5)
Vaccinating pregnant heifers and cows	3 (1-5)	3 (2-4)	3 (1-5)
Vaccination of calves within the first week of life	3 (2-5)	2 (1-5)	3 (1-5)
Vaccinating calves at spring processing (i.e. approximately 1-3 months of age)	4 (2-5)	4 (2-4)	4 (2-5)

3b. Given your experience, what vaccine would you recommend for each category of animal to reduce the incidence of fatal BRD in preweaned beef calves?

Thresholds used for colour coding responses:

Categories	Percent of respondents
LOW	≤8.3%
INTERMEDIATE	8.3-50%
HIGH	>50%

	**Vaccine of choice**	**Percent of respondents who selected the vaccine**
Vaccinating heifers and cows at pregnancy check	Intranasal modified live viral vaccine against BHV1, PIV3, and BRSV	8%
	Intranasal avirulent bacterial vaccine against MH and PH	8%
	Intranasal live viral, avirulent live culture against BHV1, BRSV, PIV3, MH and PM	0%
	Parenteral modified live viral vaccine against BHV1, PIV3, BRSV, and BVDV	50%
	Parenteral killed viral vaccines against BHV1, PIV3, BRSV, and BVDV	50%
	Parenteral bacterial toxoid vaccines against MH and PM	17%
Vaccinating heifers and cows at spring turnout	Intranasal modified live viral vaccine against BHV1, PIV3, and BRSV	25%
	Intranasal avirulent bacterial vaccines against MH and PM	8%
	Intranasal modified live viral, avirulent live culture against BHV1, BRSV, PIV3, MH and PM	8%
	Parenteral modified live viral vaccine against BHV1, PIV3, BRSV, and BVDV	75%
	Parenteral killed viral vaccines against BHV1, PIV3, BRSV, and BVDV	8%
	Bacterial toxoid vaccines against MH and PM	25%

Vaccinating pregnant heifers and cows	Intranasal modified live viral vaccine against BHV1, PIV3, and BRSV	17%
	Intranasal avirulent bacterial vaccines against MH and PM	8%
	Intranasal modified live viral, avirulent live culture against BHV1, BRSV, PIV3, MH and PM	8%
	Parenteral modified live viral vaccine against BHV1, PIV3, BRSV, and BVDV	58%
	Parenteral killed viral vaccines against BHV1, PIV3, BRSV, and BVDV	50%
	Parenteral bacterial toxoid vaccines against MH and PM	17%
Vaccinating calves within the first week of life	Intranasal modified live viral vaccine against BHV1, PIV3, and BRSV	83%
	Intranasal avirulent bacterial vaccines against MH and PM	42%
	Intranasal modified live viral, avirulent live culture against BHV1, BRSV, PIV3, MH and PM	75%
	Parenteral modified live viral vaccine against BHV1, PIV3, BRSV, and BVDV	8%
	Parenteral killed viral vaccines against BHV1, PIV3, BRSV, and BVDV	0%
	Parenteral bacterial toxoid vaccines against MH and PM	0%
Vaccinating calves at spring processing	Intranasal modified live viral vaccine against BHV1, PIV3, and BRSV	50%
	Intranasal avirulent bacterial vaccines against MH and PM	17%
	Intranasal modified live viral, avirulent live culture against BHV1, BRSV, PIV3, MH and PM	58%
	Parenteral modified live viral vaccine against BHV1, PIV3, BRSV, and BVDV	92%
	Parenteral killed viral vaccines against BHV1, PIV3, BRSV, and BVDV	8%
	Parenteral bacterial toxoid vaccines against MH and PM	58%

4. Vaccination is a disease control strategy that may be used to prevent other causes of **mortality** in pre- weaned beef calves. **What is the effectiveness, ease of implementation, and economic feasibility of vaccinating the productive groups listed below to reduce mortality in preweaned beef calves?**

	**Effectiveness** (Median, min-max)	**Ease of implementation** (Median, min-max)	**Economic feasibility** (Median, min-max)
Administering clostridial vaccines to pregnant dams	4 (0-5)	4 (1-5)	5 (1-5)
Administering clostridial vaccines in preweaned calves	5 (3-5)	3.5 (2-5)	5 (2-5)

COLOSTRUM MANAGEMENT:

5. Colostrum management is a disease control strategy that may be used to prevent **mortality** in pre- weaned beef calves. **What is the effectiveness, ease of implementation, and economic benefit of the colostrum management strategies listed below?**

	**Effectiveness** (Median, min-max)	**Ease of implementation** (Median, min-max)	**Economic feasibility** (Median, min-max)
Feeding colostrum or colostrum replacer to the calf using a nipple bottle, if it has not nursed	5 (4-5)	3.5 (2-5)	4.5 (3-5)
Feeding colostrum or colostrum replacer to the calf using an oesophageal tube, if it has not nursed	5 (3-5)	3 (2-5)	5 (3-5)

BIOSECURITY:

6. Biosecurity practices are disease control strategies that may be used to prevent **mortality** in pre- weaned beef calves. **What is the effectiveness, ease of implementation, and economic benefit of the strategies listed below?**

	**Effectiveness** (Median, min-max)	**Ease of implementation** (Median, min-max)	**Economic feasibility** (Median, min-max)
Isolating new cattle for a period of time prior to introducing them into the herd	4 (2-5)	2 (1-4)	4 (2-5)
Vaccinating new cattle prior to introducing them into the herd	4 (1-5)	3 (1-5)	4 (1-5)
Asking about the disease history of an animal or its herd prior to purchase	4 (1-5)	4 (1-5)	4 (2-5)
Testing for disease prior to introduction to the herd (e.g. BVD or Johne's)	4 (2-5)	1.5 (1-4)	2 (1-5)
Calving heifers in a separate area or at a different time than cows	5 (2-5)	2 (2-4)	4 (2-5)
Participating in certification programs (e.g. VBP+, CRSB Certified)	3 (1-5)	3 (1-4)	3 (1-4)
Other *(Please specify):* repeat purchasing from trusted sources (respondent 1O00)	4	4	3

BREEDING, CALVING, NUTRITIONAL, AND PASTURE MANAGEMENT:

7. Breeding, calving, nutritional, and pasture management are disease control strategies that may be used to prevent **mortality** in preweaned beef calves. **What is the effectiveness, ease of implementation, and economic benefit of the strategies listed below?**

	**Effectiveness** (Median, min-max)	**Ease of implementation** (Median, min-max)	**Economic feasibility** (Median, min-max)
Limiting the breeding season length of 45-60 days	4 (2-5)	3 (2-5)	4 (2-5)
Using low stocking density, such as those associated with more extensive systems	4 (2-5)	2.5 (2-4)	2 (2-4)
Using an intensive pasture rotation during calving, such as the sandhills or foothills calving systems	5 (3-5)	2.5 (1-3)	3 (2-5)
Moving cow herd from wintering area to a clean calving area	4.5 (3-5)	3 (2-4)	3.5 (2-5)
Having a creep calf area for calves (e.g. creep feeding, calf shelters)	4 (2-5)	3 (2-4)	3 (2-5)
Forced feeding mineral supplementation to cows or heifers (e.g. mixing into feed)	4.5 (2-5)	2 (1-4)	3.5 (2-5)
Injectable mineral supplementation given to cows or heifers	3 (1-5)	2.5 (2-4)	2.5 (1-4)
Injectable or oral mineral supplementation given to calves	3 (1-5)	3 (2-4)	3 (1-4)

ANTIBIOTIC ADMINISTRATION:

8. Antibiotic administration may be used as a disease control strategy to prevent **mortality** in preweaned beef calves. **What is the effectiveness, ease of implementation, and economic feasibility of the strategies listed below?**

	**Effectiveness** (Median, min-max)	**Ease of implementation** (Median, min-max)	**Economic feasibility** (Median, min-max)
Prophylactic use of antibiotics	2 (0-5)	2 (1-5)	1 (0-5)
Metaphylactic use of antibiotics	2.5 (1-5)	3 (1-4)	2 (1-4)
Mass antibiotics treatment	3 (2-5)	2 (1-4)	3 (0-4)

If you wish, please provide details of when you would or would not use each of the above antibiotic administration strategies:

Respondent's comments are listed below, and each respondent has been identified using an alpha numerical code.

- There needs to be a specific diagnosis made before this is done and the antimicrobial chosen has to be effective for that disease (1A00).
- I would likely never consider a prophylactic antbiotic for any cow-calf herd. In my experience, it is quite rare I experience a scenario where bacterial infections flourish, mostly viral (whether NCD or BRD). (1D00).
- PROPHYLAXIS: RARELY USED. Metaphylaxis: used when large groups commingled. Mass Treatment: Used in Outbreaks (1K00).
- For prevention of mortality only mass medication would be a choice if more than 10% of calves get treatment for particular disease. Sick calves are more likely to die. (1M00)
- Prophylaxis would only be recommended after all other management strategies have been implemented (and they'd have to be doing something really wrong, I've never needed to). I'd actively price them out of prophylactic treatment of whole calf groups/ withhold service if they were consistently practicing prophylaxis without first amending management practices or ignoring recommendations. Metaphylactics may be used on occasions where diagnostics (postmortem exam or fecal sample) support antimicrobial treatment and >3% mortality in 1week or less or >10% exhibiting clinical disease at any given time. In both occasions it would be after examination of the group and further discussion around prevention strategies and would not be expected to be used as part of a herds routine management of disease. (1O00).

If there is a disease control strategy that you consider to be important for preventing <u>mortality</u> of pre- weaned beef calves that was not listed above, please describe it in the box below.

Respondent's comments are listed below, and each respondent has been identified using a code.

- With outbreaks, a definition has to be created for a definition of an outbreak and the disease needs to be diagnosed and one that will respond to a environmental management - cleaning pens/barns to reduce manure buildup and spread of disease. Training on disease diagnosis, treatment protocols and records. valid VCPR - herd health program, with herd specific vaccination protocols based on known disease risks.... (1A00)

- Management is key. Drugs and products are rarely the answer, only a tool in the toolbox (1D00).

- 1.Limit gathering and bunching of any kind. 2. Limit additions to group where possible (1K00).

- Segregation by age group, was mentioned in Sandhills system somewhat? (1M00)

- Postmortem examination and diagnostics of dead calves & communication with your vet & peers. - if you are not monitoring what's going and you don't know what preventative measures can be used on you can't manage appropriately (1O00).

Thank you for reading the first report. Please proceed to completing the second questionnaire

Supplementary Material S2. Feedback report of the second questionnaire in an expert consensus study regarding management practices to prevent infectious mortality in preweaned beef calves in western Canada.

This is the second report showing the responses to Questionnaire 2.

The aim of this report is to provide feedback to the experts regarding the group's responses to Questionnaire 2. This is an essential step in the Delphi method where the experts (i.e. you) can consider the responses before participating in the final workshop.

<u>How were responses analyzed?</u>

For the question that required the input of percentages (Q1), the median, minimum, and maximum values were reported.

To analyze the scores for each element (i.e., effectiveness, ease of implementation and economic feasibility) of a strategy (Q2a, Q3a, Q4, Q5, Q6, Q7, and Q8), the median, minimum, and maximum scores are reported. Then, colour coding was used to visualize median scores, as shown below.

0-0.5	0.5-<1.5	1.5-<2.5	2.5-<3.5	3.5-<4.5	4.5-5
Not at all for any herd	Not at all for most herds	Somewhat for some herds	Very much for some herds	Very much for most herds	Always for all herds

To analyze questions that required experts to select a vaccine of choice (Q2b and Q3b), the percentage of respondents who selected each vaccine was calculated. Thresholds used to classify 'High', 'Intermediate', and 'Low' values were determined according to the 25th and 75th percentiles of the cumulative percent of responses obtained in this questionnaire. Colour coding was used to visualize 'High' (green), 'Intermediate' (yellow), and 'Low' (red) percentages.

Finally, overall median scores for each strategy were calculated. These were estimated by using the median percentage of the relative weights given to the effectiveness, ease of implementation, and economic benefit of a general strategy (Q1) times the median scores for effectiveness, ease of implementation and economic benefit for a given strategy. Overall scores median scores will be ranked in descending order, and those with an overall median score ≥2.5 will be discussed during the final workshop.

<u>RESULTS:</u>

1. Given your knowledge and experience, what is the relative weight that should be given to the following <u>elements</u> of a disease control strategy in general terms?

	Median % (min-max)
Effectiveness	50 (35-60)
Ease of implementation	25 (15-30)
Economic feasibility	25 (10-40)

VACCINATION:

2a: Vaccination is a disease control strategy that can be used to prevent fatal NCD in pre-weaned beef calves. **What is the effectiveness, ease of implementation, and economic feasibility of vaccinating each of the production groups listed below to reduce the incidence of fatal NCD?**

	**Effectiveness** (Median, min-max)	**Ease of implementation** (Median, min-max)	**Economic feasibility** (Median, min-max)
Parenteral vaccine given to calves during the first week of life (e.g. Bovine Rotavirus and Coronavirus modified live virus)	2 (1-2)	2 (1-3)	2 (1-3)
Parenteral vaccines given to pregnant heifers following label instructions	3 (2-4)	3 (2-4)	3 (2-4)
Parenteral vaccines given to pregnant cows following label instructions	3 (3-4)	3 (2-4)	3 (2-4)

2b. Given your experience, what vaccine would you recommend for pregnant heifers and cows prior to the calving season to reduce the incidence of fatal NCD in preweaned beef calves?

Thresholds used for colour coding responses:

Categories	Percent of respondents
LOW	≤9%
INTERMEDIATE	9-57%
HIGH	>57%

	Vaccine of choice	Percent of respondents who selected the vaccine
Parenteral vaccines given to pregnant heifers	Modified live Bovine Rotavirus and Coronavirus vaccine	9%
	Killed Bovine Rotavirus and Coronavirus	18%
	Killed Bovine Rotavirus and Coronavirus vaccine with *Clostridium perfringens* Type C & D and *Escherichia coli* bacterin-toxoid	91%
	Bacterin toxoid *Clostridium chauvoei*, *C. septicum*, *C. haemolyticum*, *C. novyi*, *C. sordellii*, *C. perfringens* Types C & D	45%
Parenteral vaccines given to pregnant cows	Modified live Bovine Rotavirus and Coronavirus vaccine	9%
	Killed Bovine Rotavirus and Coronavirus	9%
	Killed Bovine Rotavirus and Coronavirus vaccine with *Clostridium perfringens* Type C & D and *Escherichia coli* bacterin-toxoid	91%
	Bacterin toxoid *Clostridium chauvoei*, *C. septicum*, *C. haemolyticum*, *C. novyi*, *C. sordellii*, *C. perfringens* Types C & D	45%

3a. Vaccination is a disease control strategy that can be used to prevent fatal BRD in pre-weaned beef calves. **What is the effectiveness, ease of implementation, and economic feasibility of vaccinating the production groups listed below to reduce the incidence of fatal BRD in calves?**

	**Effectiveness** (Median, min-max)	**Ease of implementation** (Median, min-max)	**Economic feasibility** (Median, min-max)
Vaccinating heifers and cows at pregnancy check	2 (1-5)	4 (2-4)	3 (1-5)
Vaccinating heifers and cows at spring turnout	2 (0-5)	2 (1-3)	2 (0-5)
Vaccinating pregnant heifers and cows	3 (1-5)	3 (1-4)	2 (1-5)
Vaccination of calves within the first week of life	3 (1-5)	2 (1-4)	3 (1-5)
Vaccinating calves at spring processing (i.e. approximately 1-3 months of age)	4 (3-5)	3 (2-4)	4 (3-5)

3b. Given your experience, what vaccine would you recommend for each category of animal to reduce the incidence of fatal BRD in preweaned beef calves?

Thresholds used for colour coding responses:

Categories	Percent of respondents
LOW	≤2%
INTERMEDIATE	2-70%
HIGH	>70%

	Vaccine of choice	Percent of respondents who selected the vaccine
Vaccinating pregnant heifers and cows	Intranasal modified live viral vaccine against BHV1, PIV3, and BRSV	9%
	Intranasal avirulent bacterial vaccine against MH and PH	0%
	Intranasal live viral, avirulent live culture against BHV1, BRSV, PIV3, MH and PM	0%
	Parenteral modified live viral vaccine against BHV1, PIV3, BRSV, and BVDV	82%
	Parenteral killed viral vaccines against BHV1, PIV3, BRSV, and BVDV	64%
	Parenteral bacterial toxoid vaccines against MH and PM	9%
Vaccinating calves within the first week of life	Intranasal modified live viral vaccine against BHV1, PIV3, and BRSV	91%
	Intranasal avirulent bacterial vaccines against MH and PM	55%
	Intranasal modified live viral, avirulent live culture against BHV1, BRSV, PIV3, MH and PM	64%
	Parenteral modified live viral vaccine against BHV1, PIV3, BRSV, and BVDV	0%
	Parenteral killed viral vaccines against BHV1, PIV3, BRSV, and BVDV	0%
	Bacterial toxoid vaccines against MH and PM	0%
Vaccinating calves at spring processing	Intranasal modified live viral vaccine against BHV1, PIV3, and BRSV	73%
	Intranasal avirulent bacterial vaccines against MH and PM	36%
	Intranasal modified live viral, avirulent live culture against BHV1, BRSV, PIV3, MH and PM	73%
	Parenteral modified live viral vaccine against BHV1, PIV3, BRSV, and BVDV	82%
	Parenteral killed viral vaccines against BHV1, PIV3, BRSV, and BVDV	27%
	Parenteral bacterial toxoid vaccines against MH and PM	55%

4. Vaccination is a disease control strategy that may be used to prevent other causes of **mortality** in pre-weaned beef calves. **What is the effectiveness, ease of implementation, and economic feasibility of vaccinating the productive groups listed below to reduce mortality in preweaned beef calves?**

	**Effectiveness** (Median, min-max)	**Ease of implementation** (Median, min-max)	**Economic feasibility** (Median, min-max)
Administering clostridial vaccines to pregnant dams	4 (2-5)	4 (3-5)	5 (3-5)
Administering clostridial vaccines in preweaned calves	5 (3-5)	4 (3-5)	5 (4-5)

COLOSTRUM MANAGEMENT:

5. Colostrum management is a disease control strategy that may be used to prevent **mortality** in pre- weaned beef calves. **What is the effectiveness, ease of implementation, and economic benefit of the colostrum management strategies listed below?**

	**Effectiveness** (Median, min-max)	**Ease of implementation** (Median, min-max)	**Economic feasibility** (Median, min-max)
Feeding colostrum or colostrum replacer to the calf using a nipple bottle, if it has not nursed	5 (3-5)	3 (2-4)	5 (3-5)
Feeding colostrum or colostrum replacer to the calf using an oesophageal tube, if it has not nursed	5 (3-5)	3 (2-5)	5 (3-5)
Other *(Please specify):* *Assumed calf is less than 24 hrs old above (1C00)* *Choose good mothers that nurse quickly (1K00)*			

BIOSECURITY:

6. Biosecurity practices are disease control strategies that may be used to prevent **mortality** in pre- weaned beef calves. **What is the effectiveness, ease of implementation, and economic benefit of the strategies listed below?**

	**Effectiveness** (Median, min-max)	**Ease of implementation** (Median, min-max)	**Economic feasibility** (Median, min-max)
Isolating new cattle for a period of time prior to introducing them into the herd	4 (2-5)	2 (1-3)	4 (2-5)
Vaccinating new cattle prior to introducing them into the herd	2 (3-5)	4 (2-4)	4 (3-5)
Asking about the disease history of an animal or its herd prior to purchase	3 (2-5)	4 (1-5)	4 (2-5)
Testing for disease prior to introduction to the herd (e.g. BVD or Johne's)	3 (2-4)	2 (1-4)	2 (1-4)
Calving heifers in a separate area or at a different time than cows	5 (2-5)	2 (2-4)	4 (2-5)
Participating in certification programs (e.g. VBP+, CRSB Certified)	2.5 (1-4)	2.5 (2-4)	3 (2-4)
Repeat purchasing from trusted sources	4 (3-5)	4 (2-4)	3 (3-4)
Limiting the number of purchased animals	3.5 (2-4)	2.5 (2-4)	3 (2-4)
Limiting the gathering and bunching of the herd	4 (1-5)	2 (1-4)	3 (1-4)

BREEDING, CALVING, NUTRITIONAL, AND PASTURE MANAGEMENT:

7. Breeding, calving, nutritional, and pasture management are disease control strategies that may be used to prevent **mortality** in pre-weaned beef calves. **What is the effectiveness, ease of implementation, and economic benefit of the strategies listed below?**

	**Effectiveness** (Median, min-max)	**Ease of implementation** (Median, min-max)	**Economic feasibility** (Median, min-max)
Limiting the breeding season length of 45-60 days	4 (2-5)	3 (2-4)	4 (2-5)
Using low stocking density, such as those associated with more extensive systems	4 (2-5)	2 (2-3)	2 (2-4)
Segregating calves by age using pasture management such as the sandhills or foothills calving systems	4 (3-5)	3 (2-4)	4 (2-5)
Moving cow herd from wintering area to a clean calving area	4 (3-5)	3 (2-5)	4 (3-5)
Having a creep calf area for calves (e.g. creep feeding, calf shelters)	4 (1-4)	3 (3-4)	3 (2-4)
Forced feeding mineral supplementation to cows or heifers (e.g. mixing into feed)	5 (3-5)	2 (2-4)	4 (2-5)
Injectable mineral supplementation given to cows or heifers	3 (1-4)	2.5 (2-5)	2.5 (1-4)
Injectable or oral mineral supplementation given to calves	3 (1-4)	2.5 (2-4)	3 (1-3)

ANTIBIOTIC ADMINISTRATION:

8. Antibiotic administration may be used as a disease control strategy to prevent **mortality** in pre-weaned beef calves. **What is the effectiveness, ease of implementation, and economic feasibility of the strategies listed below?**

	**Effectiveness** (Median, min-max)	**Ease of implementation** (Median, min-max)	**Economic feasibility** (Median, min-max)
Prophylactic use of antibiotics	2 (0-3)	2 (2-3)	1 (0-3)
Metaphylactic use of antibiotics	2.5 (1-4)	2 (2-3)	2 (1-3)
Mass antibiotics treatment	3 (2-4)	2 (2-3)	3 (2-3)

If you wish, please provide details of when you would or would not use each of the above antibiotic administration strategies:

Respondent's comments are listed below, and each respondent has been identified using an alpha numerical code.

- I would echo the comments of others: There needs to be a specific diagnosis made before this is done and the antimicrobial chosen has to be effective for that disease. I don't recommend the use of antibiotics for prophylaxis; occasionally antibiotics are given via metaphylaxis or mass-medication during an outbreak (1C00).
- In some situations with calving in a barn that is crowded or contaminated, I will use antibiotics to help prevent navel infections in newborns. At times will mass medicate calves in a pneumonia outbreak. If shipping valuable calves a long way in adverse weather will also sometimes use prophylactic antibiotics (1N00).
- Question 8 rarely comes up on the ranch. For example, one might use prophylactic AB use if castrating calves surgically in a dirty pen. The other two categories are only considered when confronted with significant commingling from many sources (1K00).

If there is a disease control strategy that you consider to be important for preventing mortality of preweaned beef calves that was not listed above, please describe it in the box below.

- Management is key. Post-mortem examinations are also invaluable (1C00).
- Limit the length of parturition to minimize "dumb" calves that need to be colostrum supplemented (1K00).

Ranking of overall scores of all strategies assessed during this questionnaire.

Intervention	Overall median score
Administering clostridial vaccines in preweaned calves	4.75
Feeding colostrum or colostrum replacer to the calf using a nipple bottle, if it has not nursed	4.5
Feeding colostrum or colostrum replacer to the calf using an oesophageal tube, if it has not nursed	4.5
Administering clostridial vaccines to pregnant dams	4.25
Asking about the disease history of an animal or its herd prior to purchase	4
Calving heifers in a separate area or at a different time than cows	4
Forced feeding mineral supplementation to cows or heifers (e.g. mixing into feed)	4
Vaccinating calves at spring processing (i.e. approximately 1-3 months of age) Fatal BRD<br>Vaccines with a high percentage of preference<br>• Parenteral modified live viral vaccine against BHV1, PIV3, BRSV, and BVDV<br>• Intranasal modified live viral vaccine against BHV1, PIV3, and BRSV<br>• Intranasal modified live viral, avirulent live culture against BHV1, BRSV, PIV3, MH and PM	3.75
Vaccinating new cattle prior to introducing them into the herd	3.75
Repeat purchasing from trusted sources	3.75
Limiting the breeding season length of 45-60 days	3.75
Segregating calves by age using pasture management such as the sandhills or foothills calving systems	3.75
Moving cow herd from wintering area to a clean calving area	3.75
Isolating new cattle for a period of time prior to introducing them into the herd	3.5
Having a creep calf area for calves (e.g. creep feeding, calf shelters)	3.5
Limiting the gathering and bunching of the herd	3.25
Limiting the number of purchased animals	3.125
Parenteral vaccines given to pregnant heifers following label instructions (Fatal NCD)<br><br>Vaccine with a high percentage of preference<br>• Killed Bovine Rotavirus and Coronavirus vaccine with *Clostridium perfringens* Type C & D and *Escherichia coli* bacterin-toxoid	3

Parenteral vaccines given to pregnant cows following label instructions (Fatal NCD)<br><br><u>Vaccine with a high percentage of preference</u><br>• Killed Bovine Rotavirus and Coronavirus vaccine with *Clostridium perfringens* Type C & D and *Escherichia coli* bacterin-toxoid	3
Using low stocking density, such as those associated with more extensive systems	3
Injectable or oral mineral supplementation given to calves	2.875
Vaccinating heifers and cows at pregnancy check (Fatal BRD)	2.75
Vaccinating pregnant heifers and cows (Fatal BRD)<br><br><u>Vaccine with a high percentage of preference</u><br>• Parenteral modified live viral vaccine against BHV1, PIV3, BRSV, and BVDV	2.75
Vaccination of calves within the first week of life (Fatal BRD)<br><u>Vaccine with a high percentage of preference</u><br>• Intranasal modified live viral vaccine against BHV1, PIV3, and BRSV	2.75
Injectable mineral supplementation given to cows or heifers	2.75
Mass antibiotics treatment	2.75
Participating in certification programs (e.g. VBP+, CRSB Certified)	2.625
Testing for disease prior to introduction to the herd (e.g. BVD or Johne's)	2.5
Metaphylactic use of antibiotics	2.25
Parenteral vaccine given to calves during the first week of life (e.g. Bovine Rotavirus and Coronavirus modified live virus)	2
Vaccinating heifers and cows at spring turnout Fatal BRD	2
Prophylactic use of antibiotics	1.75

Thank you for reading the second feedback report. We hope to see you during the final workshop to discuss these strategies in more detail.
